# Towards a Machine Learning Based Situational Awareness Framework for Cybersecurity: An SDN Implementation

**DOI:** 10.3390/s21144939

**Published:** 2021-07-20

**Authors:** Yannis Nikoloudakis, Ioannis Kefaloukos, Stylianos Klados, Spyros Panagiotakis, Evangelos Pallis, Charalabos Skianis, Evangelos K. Markakis

**Affiliations:** 1 Department of Information & Communications Systems Engineering, University of the Aegean, Neo Karlovasi, 83200 Samos, Greece; gnikoloudakis@aegean.gr (Y.N.); cskianis@aegean.gr (C.S.); 2 Electrical and Computer Engineering Department, Hellenic Mediterranean University, Herakleion, 71410 Crete, Greece; s.klados@pasiphae.eu (S.K.); spanag@hmu.gr (S.P.); pallis@pasiphae.eu (E.P.); markakis@pasiphae.eu (E.K.M.)

**Keywords:** situational awareness, intrusion detection systems, vulnerability assessment, machine learning, SDN, software defined networking

## Abstract

The ever-increasing number of internet-connected devices, along with the continuous evolution of cyber-attacks, in terms of volume and ingenuity, has led to a widened cyber-threat landscape, rendering infrastructures prone to malicious attacks. Towards addressing systems’ vulnerabilities and alleviating the impact of these threats, this paper presents a machine learning based situational awareness framework that detects existing and newly introduced network-enabled entities, utilizing the real-time awareness feature provided by the SDN paradigm, assesses them against known vulnerabilities, and assigns them to a connectivity-appropriate network slice. The assessed entities are continuously monitored by an ML-based IDS, which is trained with an enhanced dataset. Our endeavor aims to demonstrate that a neural network, trained with heterogeneous data stemming from the operational environment (common vulnerability enumeration IDs that correlate attacks with existing vulnerabilities), can achieve more accurate prediction rates than a conventional one, thus addressing some aspects of the situational awareness paradigm. The proposed framework was evaluated within a real-life environment and the results revealed an increase of more than 4% in the overall prediction accuracy.

## 1. Introduction

According to a recent European’s Union Agency for Cybersecurity (ENISA) report [1], a 54% increase was reported in the total number of data breaches by midyear 2019, compared to 2018. In addition, 71% of the reported data breaches were financially motivated. Additionally, according to ENISA’s “Threat Landscape 2020—Information Leakage” report [2], organizations experienced an 11% increase in disclosures compared with 2018, while in 2019 there were 2.013 confirmed data disclosures [3].

One of the most common ways to handle cyber-attacks, as identified by ENISA, is Intrusion Detection Systems (IDS) [4,5,6], but their limitations impair their effectiveness against several malicious activities. One major limitation is that most of these systems utilize only a certain type of data as input (e.g., NetFlow v5 data) to perform their predictions/detections. The complexity and heterogeneity of current infrastructures render these systems obsolete since, to achieve a holistic operational environment awareness and provide more accurate predictions, the ingestion of diverse data, gathered from various sources, is required.

The situational awareness (SA) paradigm seems to be a very promising approach in the cybersecurity domain since it dictates the collection, fusion, and assessment of heterogeneous information from the operational environment to make predictions about possible risks, such as cyber-attacks. In more detail, according to Endsley et al. [7], situational awareness is the ability to gather data from the surrounding environment, capture temporal and spatial incidents, assess their significance, and make predictions on their forthcoming condition. While these processes were initially identified for military applications, Bass Tim [8,9] first stated that the future of cybersecurity lies in the application of the situational awareness theorem. He recommended that the next generation of intrusion detection systems should fuse data from heterogeneous sources for the creation of cyberspace situational awareness.

In consideration of the above, situational awareness in cybersecurity allows network administrators and security analysts to gather heterogeneous data, such as network traffic data and discovered vulnerabilities, to gain a more thorough understanding of the surrounding environment. On top of that, the interpretation of that information provides insight and knowledge of the network, while assisting in the predictions about the foreseeable future [10].

Situational awareness within the cybersecurity domain has been approached in various ways in the literature [11]. Some authors propose novel data-fusion methods for heterogeneous data, while others propose machine learning (ML) techniques, to automate the SA assessment process.

Even though all those approaches propose novel exploitation methods for the situational awareness paradigm in the cybersecurity domain, they only address specific steps of the SA process, such as data collection and analysis. Moreover, they offer semi-automated or completely manual solutions, thus requiring human interaction to a great extent. Finally, towards addressing these issues, this paper introduces an automated situational awareness for cybersecurity framework that collects and assesses hosts’ environmental data (namely real-time network flows) along with discovered vulnerabilities (vulnerability assessment results [12]), to predict imminent cyber-attacks on network-enabled assets (entities). In more detail, the proposed framework monitors, detects, and assesses existing and newly introduced network-enabled entities against tens of thousands of well-known reported vulnerabilities. It produces a classification score based on the standardized Common Vulnerability Scoring System (CVSS V3.1) [13] and finally assigns each entity to a connectivity-appropriate layer 3 network slice, by utilizing the SDN controller. Thereafter, an ML-based IDS, trained with an enhanced dataset (network flows and common vulnerabilities and exposures—CVEs identifiers), continuously monitors the assessed entities, and detects ongoing attacks, by fusing, in real-time, their network flows and the vulnerability assessment results of each host.

The results of our evaluation experiments illustrated that the proposed ML-based enhanced IDS is more efficient by more than 4% in terms of prediction accuracy, compared to typical ML-based IDS, which is solely trained with network data.

The rest of this paper is structured as follows. Section 2 presents the state of the art regarding vulnerability assessment, intrusion detection systems, and situational awareness in the ICT domain. Section 3 presents the implementation details of the proposed framework. Section 4 elaborates on the performance evaluation procedure and the experimental results. Finally, Section 5 concludes this paper with a brief discussion on the outcomes of this research and the presentation of the foreseen future steps.

## 2. State of the Art

Several research initiatives and studies have been conducted concerning the utilization of the situational awareness paradigm within the cybersecurity domain. In this section, we will present the most prevalent ones that outline the current landscape.

As described by NIST [14], the cybersecurity lifecycle consists of five discrete steps, namely, Identify, Protect, Detect, Respond, and Recover. Most of the existing research endeavors focus only on one of those steps, while the remaining four most of the time are either not handled, or addressed in a non-automated manner.

Within the context of risk identification, (first step—Identify), S. Lee et al. proposed a security assessment framework, specifically designed for software defined networking (SDN) [15]. The framework utilizes the SDN paradigm to identify entities within its infrastructure. Consequently, the framework produces attack scenarios and initiates security assessment procedures at the discovered entities within the underlying network, using penetration tests.

In addition, techniques of automated random data generation for input (fuzzing techniques) are deployed to detect potential unknown attack scenarios. Similarly, Nikoloudakis et al., in [16] leveraging the SDN paradigm, developed a vulnerability assessment framework for private networks, wherein it automatically assesses newly introduced and existing network-enabled entities against known vulnerabilities. Furthermore, the proposed framework, as a protection action assigns the network-enabled entities to a connectivity-appropriate VLAN according to their vulnerability status (CVSS score). F. Loi et al. [17] proposed a suite consisting of security tests. The security tests entail assessments on (i) Confidentiality (whether the data is in plaintext, encoded, or encrypted), (ii) Integrity (checks for replay attacks and DNS security), (iii) Access Control and Availability (DoS attacks), and (iv) Reflection (malformed packets that send ICMP messages, SSDP broadcasts, and SNMP requests). Furthermore, taking public networks into consideration, E. T. Tchao et al. [18] presented an assessment framework that was evaluated on a University campus, using the Bring Your Own Device (BYOD) paradigm. In their paper, they proposed a multi-faceted authentication model to recognize patterns and usual threats to alert the network administrator. A solid contribution for security enforcement in the IoT domain, IoT Sentinel, was proposed by M. Miettinen et al. in [19]. IoT Sentinel restricts communications between vulnerable devices and attackers. It identifies the devices’ types and uses a vulnerability database to discover their vulnerabilities. In contradiction to our proposed framework, most of the aforementioned research endeavors focus only on the Identify section of the cybersecurity lifecycle, and most of them even refrain from taking any action after the assessment.

Some research initiatives have approached situational awareness with the use of machine learning. A recurrent neural network (RNN) was developed for an anomaly-based IDS from Li et al. [20]. The RNN is trained to identify network anomalies and enforce control policies, which by utilizing the benefits of the SDN paradigm. X. Liu et al. in [21,22] presented a prototype of a multiclass support vector machine (SVM)-based fusion engine. Their outcomes suggest that the SVMs have potential in real-time IDS applications, due to the faster results and reliability of the SVM approach. Additionally, Thaseen et al. [23] proposed a random tree model, reaching an increase in prediction accuracy and reducing the false alarm rate, whereas Zhang et al. [24] proposed data augmentation using a generative adversarial network (GAN). The GAN model generated data similar to the KDD99 dataset [25]. This dataset allowed the detection model to identify not only attacks, but attack variants as well. These initiatives, apart from utilizing outdated datasets, also refrain from fusing other kinds of data and only ingest and assess network data.

Finally, several studies have tried to approach cyber-situational awareness by fusing information from the operational environment. More specifically. M. L. Matthews et al. in [26] presented a cooperative procedure to achieve situational awareness in a cybersecurity context. Their system comprised a collaborative situation-aware intrusion detection system (IDS) that collects data from separate sensors. The data aggregation is performed with the help of a custom ontology, specifically designed for the fusion of heterogeneous data, resulting in a knowledge base. A recent approach from Y. Zhang et al. [27] presented a visual analysis framework that provides cyber-situational awareness. Their proposed framework provides visual analysis of the network topology. Moreover, with dynamic queries, the security analyst can filter out specific network features that are shown. In addition to those approaches, H. Park et al. [28] suggested an alternative course of action for cyber-situational awareness by combining regular expressions and their own proposed evaluation methodology to overcome the limited string-based matching that most signature-pattern matching algorithms use. The collection of the regular expressions that create detection rules is expandable and coordinated by each security analyst who utilizes the framework.

E. Dynikova et al. [29] addressed cyber-situational awareness with a mathematical/probabilistic approach. They applied risk assessment techniques to calculate attack risk levels for a specific entity and offer some countermeasures for individual attack sequences. Similarly, H. Wang et al. in [30] utilized a quantification method to enhance cyber-situational awareness. Analytic hierarchy process [31,32], along with D-S evidence theory [33], was utilized to fuse multi-source data, and eventually streamline the cyber-situational assessment process. Finally, T. Jirsik et al. in [34,35] approached situational awareness in a more traditional manner. They introduced a new and enhanced way to analyze network flows by taking advantage of the integrated meta-data that IPFIX [36] provides, resulting in a more detailed understanding of the network while eliminating the need for extra parsers, like syslog, due to the extra, non-network data from IPFIX.

The aforementioned initiatives approach situational awareness by fusing data from heterogeneous sources. Nevertheless, all of them refrain from utilizing machine learning techniques to assist in the assessment and prediction process.

As our literature review revealed, some case studies, even though they suggest approaches that include the use of machine learning algorithms, utilize outdated and possibly obsolete training datasets to identify malicious traffic, while others that fuse heterogeneous data to achieve situational awareness abstain from utilizing machine learning techniques, thus limiting the potential of their framework. Furthermore, to the best of our knowledge, the aforementioned initiatives require human intervention to a great extent, thus limiting the responsiveness of their systems.

To address these issues, we present a framework that builds upon the notion of situational awareness, and provides automation in several steps of the cybersecurity lifecycle, thus diminishing human interaction. In more detail, the proposed framework:

- Discover existing and newly introduced network-enabled entities in real-time by utilizing the SDN paradigm.
- Performs vulnerability assessment on discovered network-enabled entities using a Vulnerability Assessment as a Service (VAaaS) component.
- Assigns the assessed entities to a connectivity-appropriate network slice through an SDN controller application, depending on their risk level (vulnerability assessment).
- Leverages an enhanced dataset, which combines heterogeneous data to train an ML-based IDS, thus achieving more fine-grained classification, and therefore more robust training results.
- Produce intrusion detection predictions, by utilizing real-time data (Netflow data and system vulnerabilities—CVEs).

## 3. System Architecture

**Figure 1:**
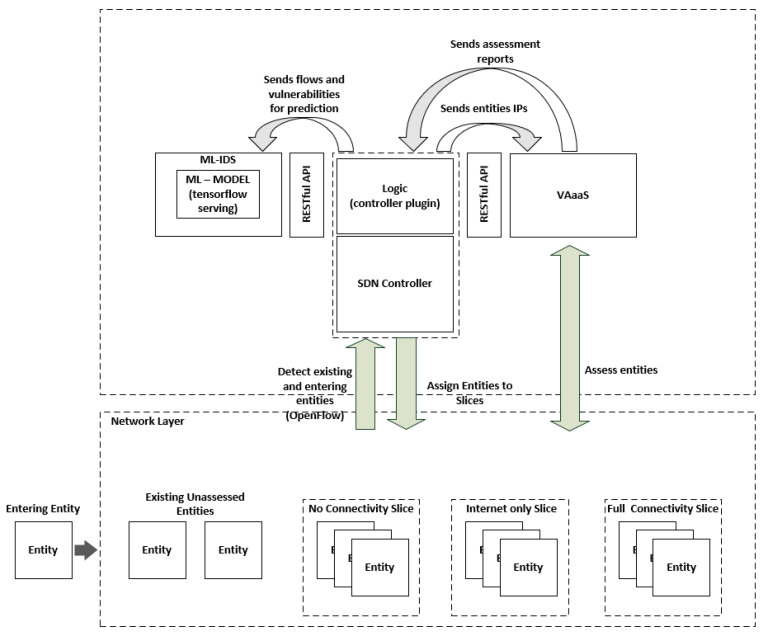
System architecture.

This section presents the high-level architecture of the proposed framework and elaborates on all internal components and their functionalities (Figure 1). Moreover, the framework’s overall functionality is described by two basic use cases, which relate to steps 1, 2 and 3 of NIST’s cybersecurity lifecycle [37], namely Identify, Protect and Detect, which are also presented in the following subsections.

### 3.1. Logic Component

The logic component is an over-the-top SDN controller application that facilitates the overall functionalities of the presented framework. Inherently, the logic component is aware of its underlying network topology, in real-time. It instructs the VAaaS component to perform vulnerability assessments on newly introduced entities upon discovery (discovery is performed in real-time). Moreover, previously assessed entities are periodically reassessed in a preconfigured ad hoc manner. Based on the risk level that derives from the vulnerability assessment report (CVSS score), each entity is assigned to a connectivity-appropriate network slice. There are three distinct network slices that represent the entities’ risk-levels. Namely, the “full connectivity” slice, for low risk level (CVSS between 0.1–3.9), the “internet-only” slice for medium risk level (CVSS between 4.0–6.9), and “no-connectivity” slice for high and critical risk level CVSS between 7.0–10.0). Thus, entities with high-risk levels (CVSS score above 7.0 [38]), posing a potential threat to the network, will not be granted network access. The slicing is dynamically enforced by applying all the necessary flow-rules for the target entity on the SDN controller, by the logic component.

Moreover, the logic component also facilitates the fusion and propagation of the appropriate data to the ML-IDS. In more detail, it collects the NetFlow data from the entities, fuses them with the discovered vulnerabilities, if any, for each entity, and sends them to the ML-IDS. In this way, the ML-IDS can perform its prediction. More structured details concerning the functionalities of the component will be presented in the following subsections.

### 3.2. Vulnerability Assessment as a Service (VAaaS) Component

The VAaaS component exposes a RESTful API, allowing for the programmatic manipulation of its internal functionalities. Therefore, as explained above, the VAaaS component performs vulnerability assessments on each entity the Logic component dictates, and produces an assessment report, containing the discovered vulnerabilities and the overall risk level (CVSS score [39]) of the assessed entity. This report will be used by the Logic component to assign each entity to a connectivity-appropriate network slice. Additionally, the discovered vulnerabilities will be fused with the real-time NetFlow data to be fed to the ML-IDS.

### 3.3. ML-IDS

#### 3.3.1. Dataset

For the purposes of the evaluation of this research we created two (2) datasets, plain and enhanced, using the features comprised by the CIC-IDS2017 dataset [40] and the data captured from real-life network traffic, within our evaluation environment. In more detail, the plain dataset is similar to the CIC-IDS2017 format, while the enhanced dataset also contains common vulnerabilities and exposures (CVEs) definitions as extra features. Finally, each entry in both datasets were labeled with the name of the specific attack.

#### 3.3.2. ML-Model

The continuous monitoring of the underlying network is performed by the ML-IDS. The ML-IDS component is an intrusion detection system that utilizes a neural network (NN) to accurately detect imminent cyber-attacks. The component’s ML model has been trained with our enhanced dataset. Based on the fact that we have a multiclass classification problem, and according to the literature [41,42,43,44], for the creation of our neural model, we initiated a trial and error procedure, using the Rectified Linear Unit (ReLU) activation function in the hidden layers of our model. However, during the testing procedure, we observed that the Hyperbolic Tangent activation function (Tanh) illustrated a considerable increase in both training and test accuracy compared to ReLU. Therefore, the Tanh activation function was selected. For the output layer of the model, we used the SoftMax function. For the purposes of this study, and since this is a proof-of-concept framework, the dataset only included five easily reproducible attacks. The attacks that were used were Elasticsearch Remote File Inclusion Attack, Manage Engine Remote Desktop Attack, WordPress Ninja Forms Attack, OpenSSH Server Service Brute Force Attack, and Denial of Service Slow Loris Attack. The ML-IDS’s neural network is exposed through the TensorFlow Serving [45] component, by exposing a RESTful API, allowing for programmatic manipulation of the neural model.

### 3.4. Use Cases

The presented framework’s overall functionality is described by two fundamental use cases. The first use case concerns the first and second steps of the cybersecurity lifecycle, i.e., “Identify” and “Protect”. The second one concerns the third step of the cybersecurity lifecycle, “Detect”. In the second use case, we try to approach the notion of situational awareness within the cybersecurity domain by fusing heterogeneous and seemingly uncorrelated data from various sources, to gain awareness of the operational environment in real-time, and produce predictions, as accurate as possible, concerning the immediate future, e.g., predict an imminent attack on a specific entity.

#### 3.4.1. Identification and Protection Use Case

**Figure 2:**
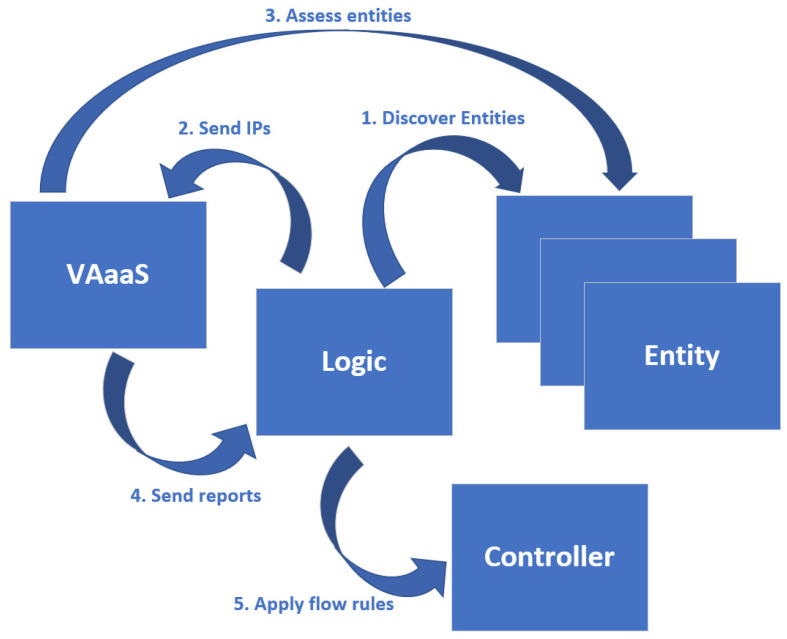
Identification use case conceptual diagram.

As briefly described in the previous subsections, the first use case involves the identification of vulnerabilities on entities and the proactive measures to protect the system from possible implications imposed by vulnerable entities, which is a rather straightforward procedure. The Logic component, since it is developed as an SDN controller application, detects newly introduced entities in real-time. Thus, upon detection, it probes the VAaaS component with the discovered entities’ IPs to initiate a vulnerability assessment on each one of them. Consequently, the VAaaS component performs the assessments, a somewhat time-consuming procedure, produces detailed reports, and sends them back to the Logic component. These reports contain a list of the discovered vulnerabilities on each entity, and the overall score of the assessment, which is based on the standardized CVSS V3.0 score and classifies the entity into one of the specified risk levels: “Critical” (9.0–10), “High” (7.0–8.9), “Medium” (4.0–6.9), and “Low” (0.1–3.9). Consequently, the Logic component receives the reports and depending on the assessment results (CVSS V3.0 score), assigns each entity to a connectivity appropriate network slice, by applying the appropriate flow-rules on the SDN controller. Three self-explanatory network slices have been developed, slice#1, no-connectivity, slice#2, internet-only, and slice#3, full-connectivity. As one can infer, the ML-IDS component does not partake in this use case. A conceptual representation of the use case is depicted in Figure 2.

#### 3.4.2. Detection Use Case

**Figure 3:**
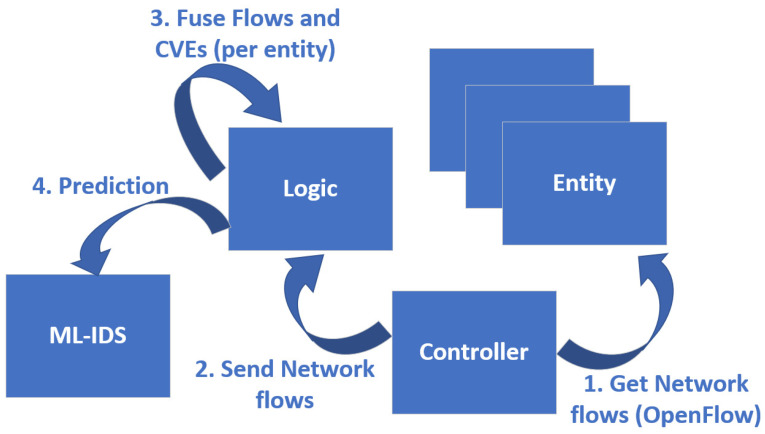
Detection use case conceptual diagram.

In this use case, the VAaaS component does not partake, since the vulnerability assessment procedure has already been completed and has provided the Logic component with the vulnerability assessment reports. Consequently, the Logic component starts collecting the network flows from each entity, through the OpenFlow protocol (packet-in). It then fuses the network data with the corresponding vulnerabilities and sends it to the ML-IDS component. Finally, the ML-IDS component produces a prediction on whether an attack is taking place on an entity, and what is the name of that attack. A conceptual representation of the use case is depicted in Figure 3.

## 4. Evaluation

To evaluate the presented framework, we performed several measurements within a real-life environment to assess the overall prediction precision (Equation (1)) of the ML-IDS, i.e., the precision of the internal neural network, which is the outcome of the enhanced custom-made dataset. In more detail, the situational awareness framework was deployed on our Lab’s premises, wherein the internal network is managed by an SDN controller. Therein, we also deployed a virtual machine with the appropriate vulnerabilities, so that we can reproduce the specific cyber-attacks, which our framework was trained to detect. The following subsections present the results of this evaluation process.

### 4.1. Aim

The scope of this evaluation is to assess the overall precision of the neural network of our proposed ML-IDS. Precision is defined as the number of correctly predicted attacks out of all the performed attacks, which can be estimated by dividing the sum of correct predictions by the sum of total predictions, as demonstrated in Equation (1).

(1) $$\mathrm{Precision}=\frac{\mathrm{Correct}\;\mathrm{Predictions}}{\mathrm{All}\;\mathrm{Predictions}}$$

### 4.2. Method

The evaluation process is a two-fold procedure. For the first phase, we assess the precision of the ML model, trained with the enhanced dataset, hereafter called “enhanced model”. For the second phase, we assess the same values for the ML model, which was trained with a typical dataset, hereafter called “plain model”.

For each phase, the Logic component gathers and fuses the real-time network traffic (network flows) for each discovered entity and its discovered vulnerabilities, into a format that the trained model accepts (NumPy array). Then, the ML-IDS produces a prediction based on the input, whether an attack is in progress, and which attack. For the purposes of demonstration, and since this is a proof-of-concept implementation, the neural network was trained for five (5) specific attacks. More specifically, an Elasticsearch-specific Remote File Inclusion (RFI) attack (CVE-2014-3120) [46] (Attack #1), a ManageEngine Desktop Central 9-specific RFI attack (CVE-2015-8249) [47] (Attack #2), a WordPress-specific RFI attack (CVE-2016-1209) [48] (Attack #3), an SSH Brute-Force attack (CVE-2001-0553) [49] (Attack #4), and finally a Slow Loris DoS attack (CVE-2007-6750) [50] (Attack #5).

### 4.3. Variables

#### 4.3.1. Dependent

The dependent variable for this evaluation experiment is the precision of each prediction. The values collected for each model were assessed and compared with each other. This allowed us to gain a more detailed assessment, of the effectiveness of each respective model.

#### 4.3.2. Independent

For this evaluation experiment, we required only one independent variable, which was the type of the attack. During the experiment, we performed all the attacks the models were trained, mentioned in the Section 4.2.

#### 4.3.3. Fixed

The fixed variables for this experiment were the total duration for each respective attack. In more detail, each attack was performed for thirty seconds.

### 4.4. Prediction

The assumption that we are trying to prove is that the enhanced model will generate a greater precision rate on an identical cyber-threat incident than the plain model.

### 4.5. Results

**Figure 4:**
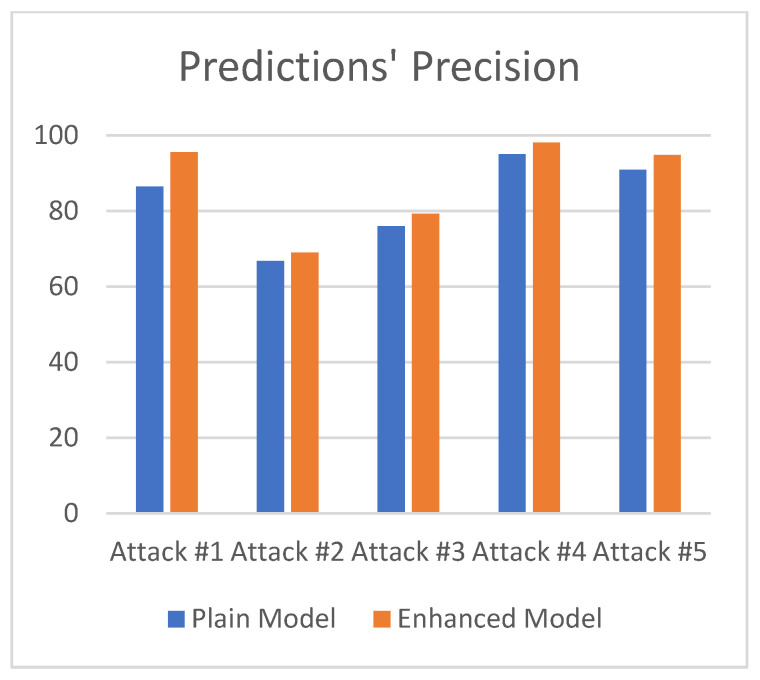
Precision of predictions for both IDSs for all attacks.

Table 1 depicts the correlation between the attacks and their attack number. Both Table 2 and Figure 4 illustrate the difference in prediction precision between the two models, while Table 3 illustrates the confusion matrix for the enhanced dataset.

### 4.6. Discussion

Both models are compared in Table 1, regarding their precision in predicting each of the attacks mentioned in the previous subsection. The first row represents the precision scores of the plain model, while the second row represents the precision values of the enhanced model.

Results signify that the enhanced model produces higher prediction accuracy results on all of the attacks. The mean precision for the plain model is ~83%, and for the enhanced model it is ~87%. The enhanced model demonstrates an increased precision of more than 4%. The graphical comparison of the two models is depicted in Figure 4.

To summarize, the enhanced model outperformed the plain model in all the attacks. Nevertheless, during the second and third attacks, even though the enhanced model correctly predicted the attack, the prediction rates for both models were rather low. This phenomenon is somewhat expected since the premise which upon both models were trained, was to classify and detect attacks based on their network footprint, meaning the deviations in network “behavior” during specific attacks. Thus, when an attack produces a small footprint, it makes it even harder for such kinds of neural models to achieve high prediction precisions.

### 4.7. Evaluation

The presented evaluation showcases and compares the variable efficiency a neural network model has on two different occasions. Firstly, when it is trained with a plain network traffic dataset, and then when it is trained with an enhanced dataset. The results were decisive for that comparison. Even so, the experiment’s variables could be modified further for even better results. From our perspective, concerning the neural network model, more algorithms could be tested, to produce even higher prediction precision. Furthermore, the enhanced model produces higher prediction precision on all of the attacks, in comparison with the plain model. However, the method utilized for the creation of that dataset could be revised, since it now allows the model to perform better, but if the number of attacks increases, it might not perform as well, since the dimensionality of the dataset will change, leading to an increase in false positives/false negatives. Further investigation is required to efficiently fuse such kind of heterogeneous data to offer scalability to the framework.

## 5. Conclusions

In this paper, we presented an automated framework that addresses the first three steps of the cybersecurity lifecycle, as defined by NIST (Identify, Protect and Detect). The presented framework performs vulnerability assessment on network-enabled entities in the network (Identify), assigns each entity to a connectivity-appropriate network slice (Protect), and finally continuously monitors the underlying infrastructure with an ML-based IDS, utilizing the situational awareness paradigm (Detect). The ML-IDS has been trained with a custom enhanced dataset that contains network data (NetFlow) and CVEs.

The framework was evaluated through a series of experiments, within a real-world environment comprising a minimum set of CVEs for proof of concept purposes. The evaluation results indicated the increased prediction performance of the presented ML-IDS in comparison with a normal ML-based IDS. Although the results look promising, we aim at testing alternative algorithms for the enhancement of our neural model, and further research and examine the dataset creation procedure. Our goal is to create a dataset containing as many attacks as possible.

## Figures and Tables

**Table 1:** Attack–attack Number Correlation.

Attack	Attack Number
Remote File Inclusion (RFI) attack	Attack #1
ManageEngine Desktop Central 9-specific RFI attack	Attack #2
WordPress-specific RFI attack	Attack #3
SSH Brute-Force attack	Attack #4
Slow Loris DoS attack	Attack #5

**Table 2:** Models’ predictions’ precision on live attacks.

Precision	Attack #1	Attack #2	Attack #3	Attack #4	Attack #5	Standard Deviation	Mean
Plain model	86.42	66.80	75.92	95.01	90.86	10.29	83.00
Enhanced Model	95.55	68.96	79.19	98.06	94.82	11.33	87.32
Delta	9.13	2.15	3.27	3.05	3.96	1.04	4.31

**Table 3:** Confusion matrix for enhanced dataset.

True/Predicted	Elastic	Manage Engine	Wordpress	SSH	Slowloris
Elastic	387	0	0	0	18
ManageEngine	0	164	0	0	20
Wordpress	0	0	288	34	0
SSH	0	0	4	1819	10
Slowloris	18	74	72	2	877

## Data Availability

Not applicable.
